# Effect of Manganese Oxide Sand Used in Water Treatment in Removing ^222^Rn and Mn^2+^, Fe^2+^_,_ NH_4_^+^, and NO_3_^−^ from Groundwater

**DOI:** 10.3390/molecules31132343

**Published:** 2026-07-03

**Authors:** Theodora-Paraschiva Gheorghe, Ileana Radulescu, Bianca-Maria Linca, Vasilica Tucureanu, Georgiana Martinica, Ion Ion, Alina C. Ion

**Affiliations:** 1 Department of Analytical Chemistry and Environmental Engineering, National University of Science and Technology Politehnica Bucharest, 060042 Bucharest, Romania; theodora.gheorghe@nipne.ro (T.-P.G.); georgiana.martinica@upb.ro (G.M.); ion.ion@upb.ro (I.I.); 2 Horia Hulubei National Institute for Physics and Nuclear Engineering—IFIN HH, SALMROM Laboratory, 077125 Magurele, Romania; rileana@nipne.ro (I.R.); bianca.linca@nipne.ro (B.-M.L.); 3 Faculty of Physics, University of Bucharest, 077125 Bucharest, Romania; 4 National Institute for Research and Development in Microtechnologies IMT-Bucharest, 077190 Bucharest, Romania; vasilica.schiopu@gmail.com

**Keywords:** radon removal, manganese oxide sand, adsorption, groundwater

## Abstract

In this study, the possibility of ^222^Rn removal through the deferrization/demanganization applied for water treatment was considered, assuming a sorption process. This study demonstrates the possible removal of dissolved radon from groundwater using manganese oxide sand (MnO_2_) employed in conventional water treatment systems. Four groundwater samples with varying chemical compositions were treated, and the simultaneous removal of ^222^Rn, Mn^2+^, Fe^2+^, NH_4_^+^, and NO_3_^−^ was evaluated. Radon activity was quantified using a Lucas scintillation cell coupled with a Pylon AB-5 system, while inorganic ions were analyzed by ion chromatography. Structural characterization of MnO_2_ sand by XRD, SEM–EDS, and FTIR confirmed a heterogeneous oxide surface favorable for adsorption. The treatment achieved average removal efficiencies of 95% for ^222^Rn, 79% for Mn^2+^, 87% for Fe^2+^, 85% for NH_4_^+^, and 73% for NO_3_^−^. Equilibrium adsorption capacities increased with initial contaminant concentrations, indicating a multi-species uptake. Importantly, radon removal was largely independent of coexisting ions. These results reveal the benefit of MnO_2_-based water treatment, offering an effective solution for simultaneous radon and inorganic contaminant mitigation.

## 1. Introduction

Groundwater represents a crucial resource for households as well as for industrial and agricultural activities worldwide. In many regions, it constitutes the only available source of water. Despite its importance, groundwater is frequently affected by contamination from inorganic pollutants. The removal of contaminants from water is a critical environmental challenge, necessitating innovative technologies and methods. Recent advancements highlight various approaches, including sorption, membrane separation, and novel purification processes, which effectively target multiple reductions in contaminants from water sources. Among the most common species are ferrous iron (Fe^2+^), manganese (Mn^2+^), and ammonium (NH_4_^+^), which often occur at concentrations that require efficient removal [1]. Elevated levels of Fe^2+^ and Mn^2+^ in groundwater may pose some health risks, including potential damage to the nervous system and the development of symptoms resembling Parkinson’s disease [2]. These ions can also give water an unpleasant taste and color. At the same time, inadequate management of different types of waste, such as fertilizers and industrial effluents, has led to a continuous increase in NH_4_^+^ in groundwater. An excessive intake of ammonium can lead to cellular ionic imbalance, convulsions, and other severe effects [2].

Water pollution has become an increasingly serious issue, particularly with regard to radioactive contaminants, too. On the one hand, among these pollutants is uranium, which plays an indispensable role in the development of nuclear energy, but also other radioactive materials, which are used in many applications, such as nuclear medicine, industrial radiography, agriculture, and research. On the other hand, elevated radon levels in groundwater have been widely reported in the literature [3]. Radon(^222^Rn) is a naturally occurring radioactive gas that comes from the decay of uranium present in soil, rocks, and water [4]. Being water-soluble, radon infiltrates groundwater sources, posing potential health risks when ingested as drinking water.

There are several techniques mentioned in the literature for the removal of contaminants, including radionuclides, such as sorption processes, reverse osmosis, membrane distillation, and ion-exchange methods. Continuous research and development are essential to enhance the effectiveness and sustainability of these removal technologies. Advanced systems like reverse osmosis and membrane separation were used to separate cesium ions from radioactive wastewaters, showing significant removal efficiency [5], but these methods can also generate secondary waste, necessitating careful management of by-products [6]. Regarding radon removal in groundwater, there are three techniques reported in the literature: physical removal, sorption, and biological treatment, which all showed a high removal efficiency of up to 99% [3]. Adsorbents, granular activated carbon, manganese oxide, zeolites, and sorbents with high surface area were considered more efficient in these techniques.

Adsorption methods are favored for their high treatment rates and established industrial applications, making them a practical choice for radionuclide remediation [7]. Despite these advancements, the complexity of radionuclide contamination and the potential for secondary waste generation remain significant challenges. Adsorption techniques used in radionuclide removal have shown efficiency not only for ^222^Rn but also for uranium capture on metal–organic frameworks (MOFs) [8] and biopolymers [9].

These methods showed very good performances in water purification processes, achieving over 85% removal of multiple radionuclides [10]. The removal of ^222^Rn through the adsorption process uses the down-flow mode in which ^222^Rn-rich water is passed through a column containing the absorbent material, which, in this study, is manganese oxide sand. The removal of ^222^Rn from water is affected mainly by the contact area between the sorbent and the solution [3].

MnO_2_, used in general and in deferrization and demanganization processes, presented good efficiency in removing contaminants and even radionuclides from water due to its high surface area, good stability at certain pH values, and its behavior in redox reactions and in ion exchange [11]. During the deferrization and demanganization processes applied in the treatment of waters for iron and manganese removal, several other inorganic species can also be removed.

Manganese dioxide not only presents selectivity for manganese ions but also for other cations, among which are several radionuclides, such as ammonium, nitrate, arsenic, and other heavy metals. The adsorption and oxidation processes can be explained by the following chemical equations [12]:

Mn^2+^ + MnO_2_·xH_2_O → MnO_2_·MnO·(x − 1)H_2_O(s) + 2H^+^ (1)

MnO_2_·MnO·(x − 1)H_2_O + 1/2O_2_ + H_2_O → 2MnO_2_·xH_2_O(s) (2)

Manganese oxide showed good properties for Ra [13], Co, Th, and U ions from aqueous solutions, especially in groundwater samples. It was already used in manganese removal from natural waters, and it seems to be able to remove other species too.

In this research, a preliminary study on ^222^Rn and Mn^2+^, Fe^2+^, NH_4_^+^, and NO_3_^−^ removal in the demanganization process applied to groundwater is presented, considering the chemical composition of the waters and other parameters such as pH and electrical conductivity.

## 2. Materials and Methods

### 2.1. Materials and Reagents

The manganese oxide sand in this study was used as received and utilized in the deferrization/demanganization tanks during the treatment of the water [14].

Nitric acid (HNO_3_) 0.1 M and 2,6-pyridinedicarboxylic acid 0.02 mM, both purchased from Sigma Aldrich, for ion chromatography, as certified solutions or prepared in the laboratory, were used as the mobile phase in ion chromatography for the analysis of the cations.

The ultrapure water was from Millipore Direct Q3 with a resistivity > 18.2 MΩ × cm (25 °C). All the reagents used in the batch experiments and for analyses were of analytical reagent grade.

### 2.2. Instruments

For radon measurements in the groundwater samples, the Pylon AB5 counting system was used with a Lucas active cell (Pylon Electronics Inc., Ottawa, ON, Canada).

For the ion-chromatography analysis, the following instruments were used: Sonic Vibracell ultrasonic processor (Sonics & Materials, Newtown, CT 06470, USA); ion chromatograph—850 professional AnCat-MCS using a dosing technique (MiPT), with a conductivity detector; cationic chromatographic column—Metrosep C4-150/4.0; pre-column—Metrosep RP 2/3.5; 850 Metrohm conductivity detector (0–15,000 µS/cm); and a Metrohm Autosampler and Dosino dosing device (Metrohm, Herisau, Switzerland).

The structural morphology of MnO_2_ sand was observed by scanning electron microscopy (SEM), FEI QUANTA 200 (FEI Company, Hillsboro, OR, USA), combined with energy-dispersive spectrometry (EDS). The crystal structure was analyzed by an X-ray diffractometer (XRD) (Bruker D8 Advance, Berlin, Germany). Fourier-transform infrared spectroscopy (FTIR) was used to study the chemical bond configurations in sand containing MnO_2_ before and after adsorption. A Tensor 27 spectrometer (Bruker Optics, Karlsruhe, Germany), equipped with a Platinum ATR accessory with a single-reflection diamond crystal, was employed. All samples were recorded after 64 scans, with a resolution of 4 cm^−1^, over a spectral range of 4000–370 cm^−1^. The spectra were acquired at room temperature and processed using OPUS 6.0 software.

### 2.3. Methods

#### 2.3.1. Determining ^222^Rn Specific Activity

Radon measurements were performed using a Lucas scintillation cell coupled with a Pylon AB-5R counting system manufactured by Pylon Electronics (Canada). This laboratory-grade portable instrument enables rapid and accurate determination of radon activity. Radon concentrations in groundwater samples were measured using the emanometry technique combined with a vacuum degassing procedure. Dissolved radon was extracted using a Pylon WG-1001 Vacuum Water Degassing System (Pylon Electronics Inc., Canada), which is specifically designed to transfer radon from aqueous samples into a detection chamber. A fixed volume of 190 mL of water was introduced into the degassing vessel connected to the WG-1001 unit. The sample, containing dissolved ^222^Rn, was circulated by an integrated vacuum pump. To prevent humidity-related interference, the extracted radon gas passed through a drying tube filled with a desiccant (dryrite), followed by a bubbler to ensure complete moisture removal before entering the detection chamber. The dried radon gas was transferred into a Lucas-type scintillation cell in a sealed chamber. Alpha particles emitted during the decay of the radon and its short-lived progeny (e.g., polonium-218) interacted with the ZnS:Ag coating of the sealed chamber, producing scintillation light pulses. These scintillations were detected and counted by a photomultiplier tube, with the count rate directly proportional to the radon activity concentration in the original water sample. The gas-filled cell was then connected to the counting system, which performed five consecutive measurements of 5 min each, resulting in a total counting time of 25 min. The mean counting rate was calculated and corrected by subtracting the background count rate to determine the radon concentration for each sample [14,15].

The activity concentration of ^222^Rn in groundwater samples was calculated using the following formula [16]:

(3) $$C_{Rn}\;=\frac{\left(C-B\right)}{F\cdot 3\cdot D\cdot S\cdot V}\cdot 37\cdot 10^{-3}$$

where

**Figure 1:**
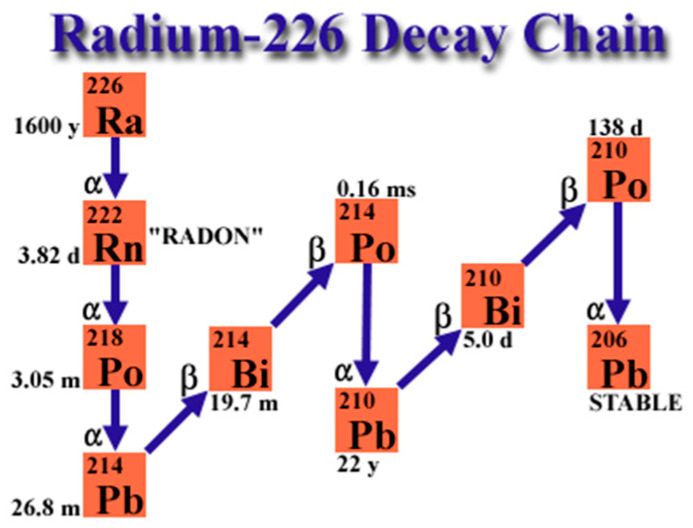
Radon (^222^Rn) decay chain [17].

C_Rn_ = ^222^Rn activity concentration in (Bq/L);
C = sample gross counting rate (in counts per minute, CPM);
B = background counting rate (in CPM);
F = cell counting efficiency (0.745);
3 = alpha-emitting steps in the radon decay chain that can be recorded, as it can be seen in the decay chain from Figure 1 (^222^Rn → ^218^Po, 1st alpha emission (the main one), ^218^Po → ^214^Pb, 2nd alpha emission, ^210^Po → ^206^Pb 3rd alpha emission);
D = degassing efficiency of the cell used (0.7);
S = time correction for the decay of radon from the sampling time Ts, between 0.6701 and 0.8217;
V = the sample volume in liters (190 mL);
37 × 10^−3^ = conversion factor between pCi and Bq.

#### 2.3.2. Chromatographic Method for Ionic Inorganic Species in Aqueous Matrices

The standard chromatographic method, according to SR EN ISO 14911:2003 [18], was used for analyzing some inorganic ions, including ammonium and manganese, from the samples.

The study uses an ion chromatograph equipped with a conductivity detector, using a column specifically designed for cation separation. The elution was performed using a mixed eluent of 3.2 mM nitric acid (HNO_3_) and 0.02 mM 2,6-pyridinedicarboxylic acid at a flow rate of 0.7 mL/min with a pressure that optimizes separation while maintaining system integrity. The system operated using a non-suppressed elution mode, since further suppression was unnecessary for improving sensitivity and resolution. The 850 Professional conductivity detector, which operates within a specific range (0–15,000 µS/cm), was used to detect the conductivity changes that occur as different cations pass through the detector. Concentrations of cations are quantified based on their retention times and the intensity of their conductivity response [19].

#### 2.3.3. Stable Phases of Manganese in Different Conditions

**Figure 2:**
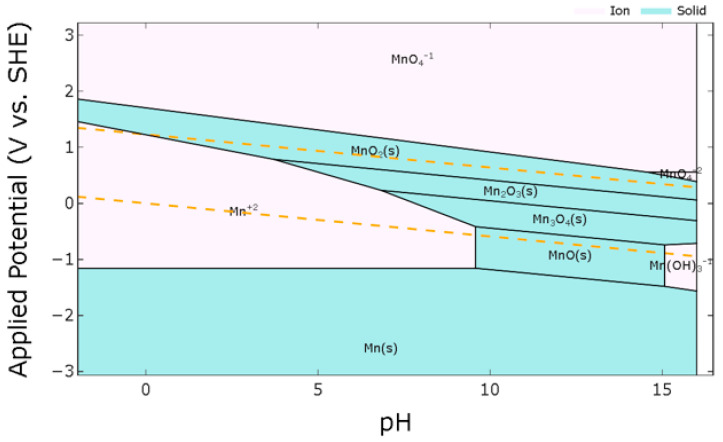
Pourbaix diagram showing the possible thermodynamically stable phases of manganese.

In order to use manganese dioxide as a sorbent in water treatment, it is important to know and understand its electrochemical behavior in various environmental conditions. The Pourbaix diagram generated and illustrated in Figure 2 shows the stable phases of manganese in different conditions, as a function of pH and electrochemical potential (Eh).

The diagram (Figure 2) divides the pH-Eh space into domains where specific manganese species are stable, including ionic forms, oxides, hydroxides, and metallic manganese. The boundaries between these species are represented by solid lines, indicating equilibrium conditions where the activities of the species are equal. The dashed lines represent the stability of water in the system [20].

#### 2.3.4. Radon Removal Experimental Removal Procedure

**Figure 3:**
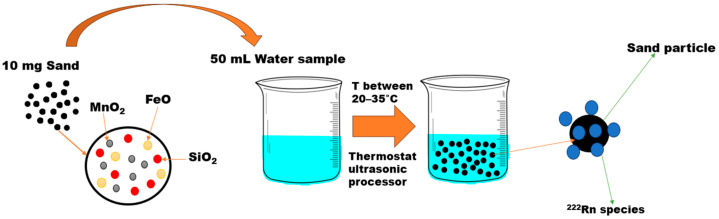
Schematic mechanism of Rn removal.

The experimental removal procedure is represented in Figure 3. Sorption static experiments for samples took place in glass flasks by adding 10 mg of MnO_2_ sand to 50 mL of water samples. The glass flasks were introduced into the thermostat ultrasonic processor, and the removal experiments took place at 25 °C. The glass material was tested before and did not present supplementary element retention. The initial pH was not modified before the experiments and was in the range of 6–8.

The equilibrium adsorption capacity (q_e_, mg/g) was considered at the attainment of the equilibrium state, after a certain experimentally verified time, and calculated with the following equation:

(4) $$q_{e}=\frac{(C_{0}-C_{e})\cdot V}{m}$$

where:

C_0_ = initial and concentrations (mg/L);
C_e_ = equilibrium concentrations (mg/L);
V = initial volume of the solution (L);
m = mass of sand(g);
q_e_ = equilibrium adsorption capacity (mg/g).

Regarding the removal percentage (RE) of Radon and the other species studied, it was calculated using the following formula:

(5) $$R_{E}\%=\frac{C_{0}-C_{e}}{C_{0}}\cdot 100$$

## 3. Results and Discussion

The ^222^Rn activity contained in groundwater is controlled by natural and anthropogenic factors like the structure of the rocks from the aquifer, rainfall infiltrations, geographical position, agricultural activities, and potable and wastewater treatment [21]. Groundwater can be used as a source of drinking water when all chemical and biological requirements are fulfilled. A permissible limit of 100 Bq/L for ^222^Rn in drinking water is indicated by the WHO (World Health Organization) [22]. ^222^Rn activity in groundwater samples is usually well below this limit, and its removal is not complicated to achieve. Therefore, in this preliminary study, the possibility of ^222^Rn removal during the deferrization/demanganization procedure applied for water treatment was monitored, assuming a sorption process.

### 3.1. Characterization Studies of MnO_2_ Sand

Manganese dioxide sand used in these studies is brown, with spherical particles, and it became thicker after contact with the aqueous solutions due to the adsorption of manganese ions from the aqueous matrices, which increases the amount of manganese oxides on the surface. For its characterization, several methods were used, among which were scanning electron microscopy and Fourier-transformed infrared spectroscopy, as described in the following sections.

#### 3.1.1. Scanning Electron Microscopy SEM

Scanning electron microscopy (SEM) was done by means of FEI QUANTA 200 (FEI Company, Hillsboro, OR, USA) combined with energy-dispersive spectrometry (EDS detector).

**Figure 4:**
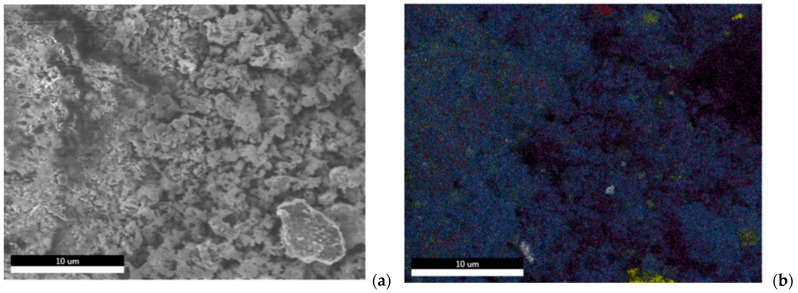
SEM images with an EDS detector of MnO_2_ sand (**a**) with element mapping of Mn (red) and Fe (yellow) for the location (**b**).

The SEM images, from Figure 4a, showed an irregularly shaped material with different sizes of grains. Its morphology is very important, considering that the uptake process of isotopes, assimilated as inorganic ionic species, is influenced by the chemical sorption and ion exchange. While Figure 4b reveals element mapping by EDS, MnOx facilitates Mn^2+^ exposed on the surface by autocatalytic oxidation, providing more adsorption sites. Compared with Mn, Fe was evenly distributed on the surface.

#### 3.1.2. Fourier Transformed Infrared Spectroscopy FTIR Analyses

**Figure 5:**
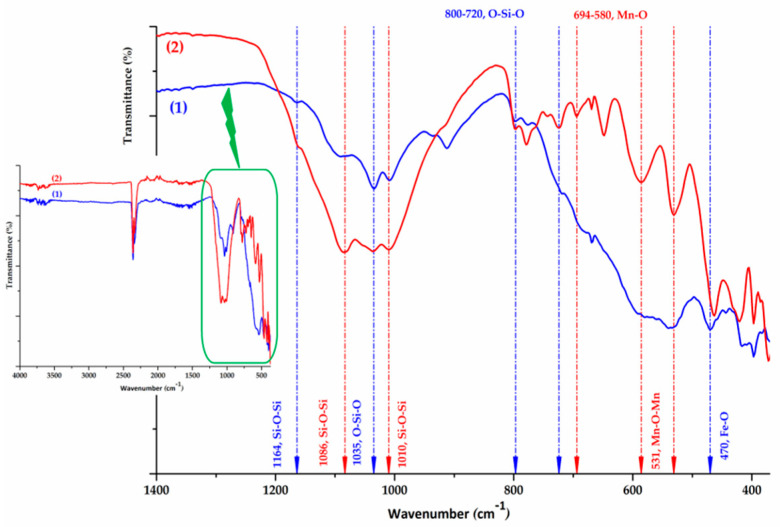
FTIR spectra of MnO_2_ sand before (1) and after (2) adsorption.

In Figure 5, the FTIR spectra recorded for manganese dioxide sand before (1) and after (2) adsorption are presented, and Table 1 indicates the possible assignments of the absorption bands.

For both samples, in the spectral range 4000–1500 cm^−1^, low-intensity bands can be distinguished, which may be associated with the vibration mode of O–H bonds from water adsorbed in the oxide material, while in the 2500–2200 cm^−1^ region, high-intensity bands appear, associated with C–O bonds from adsorbed CO_2_.

The spectral analysis highlights the presence of absorption bands that can be associated with the vibration mode of Si–O bonds, indicating the presence of SiO_2_ in the MnO_2_-containing sand. In the spectral region 1200–900 cm^−1^, bands attributed to the vibration modes of Si–O–Si bonds and to the deformation modes of O–Si–O bonds can be observed (1100–1000 cm^−1^ and 800–700 cm^−1^, respectively). For manganese sand before adsorption, the bands centered at 936 and 912 cm^−1^ may be correlated with Si–O–M bond vibrations, suggesting a possible interaction between quartz and iron and manganese oxides present in the sand. These bands decrease in intensity or even disappear after, possibly as a result of modifications in certain oxide phases or compositional differences caused by the deposition of oxide particles over the initial ones.

The presence of iron oxides is associated with bands centered at 540 and 470 cm^−1^, as well as bands below 400 cm^−1^. Considering the position and amplitude of these bands, a low Fe_2_O_3_ content in the sand can be estimated.

For both analyzed samples, the absorption bands identified in the 700–520 cm^−1^ region can be attributed to the vibration modes of Mn–O and Mn–O–Mn bonds. For the sand analyzed after adsorption, an increase in the number of absorption bands associated with Mn–O bond vibrations is observed, suggesting an increase in manganese oxide concentration in sample P2. Thus, the FTIR spectra confirm the availability of MnO_2_-specific bonds in both samples.

### 3.2. Removal Experiments

A comparison of the studied groundwater concerning its chemical composition is presented in Table 2. It can be observed that the pH values are in the range of 6–8, the electrical conductivities are between 600 and 1750 µS/cm, and the dry residue is between 278 and 1080 mg/L. High values of electrical conductivity usually indicate hard water, which needs to be softened before use by available treatments.

The concentrations of cations and anions show wide variation, as can be observed. According to the permissible limits for safe drinking water, there are no limits for calcium, magnesium, or bicarbonate. With respect to chloride, sulphate, and nitrate, the studied groundwater samples showed values below the permitted limits, meaning that if all the targeted contaminants in this study were reduced below the permitted limits, the studied groundwater could represent an important source of drinking water.

Table 3 presents the concerned anions and cations concentrations (mg/L), together with the radon concentration (Bq/L), for the studied waters, before and after the deferrization/demanganization processes. Reduced radon concentrations have been noticed, besides those of manganese and iron, after treatment. In addition, a considerable reduction has also been observed for nitrogen-containing inorganic species.

The effect of coexisting species in solution results in competitive sorption are not only seen in Mn^2+^ but also in NH_4_^+^, Fe^2+^, and ^222^Rn species. When the concentration of Fe^2+^ increases, both removal rates of Mn^2+^ and ammonium ions decrease, probably because Fe^2+^ ions occupy the sorption centers of MnO_2_ sand. ^222^Rn seems unaffected and is adsorbed on different sites on the sand surface.

It can be observed from Table 4 that MnO_2_ sand showed good sorption capacity for all the species considered in this study. The maximum values, calculated using Equation (4), were for ^222^Rn, Mn^2+^, NH_4_^+^, and NO_3_^−^ in sample 4 and were 2.18 Bq/g, 0.53 mg/g, 0.51 mg/g, and 0.65 mg/g, respectively. While for Fe^2+^, the maximum adsorption capacity was in sample 1 and was 1.45 mg/g.

#### 3.2.1_._^222^Rn Activity Concentration Before and After Demanganization Treatment

The ^222^Rn activity and the chemical composition of groundwater depend on both natural and anthropogenic factors, including the geological structure, the lithology, and the depth of the aquifer, as well as human activities such as agriculture, industry, and water or wastewater management. For the analyzed samples, the ^222^Rn levels appear to be primarily associated with the characteristics of the aquifer rocks and structural setting, while additional contributions may arise from rainwater infiltration and other human influences.

**Figure 6:**
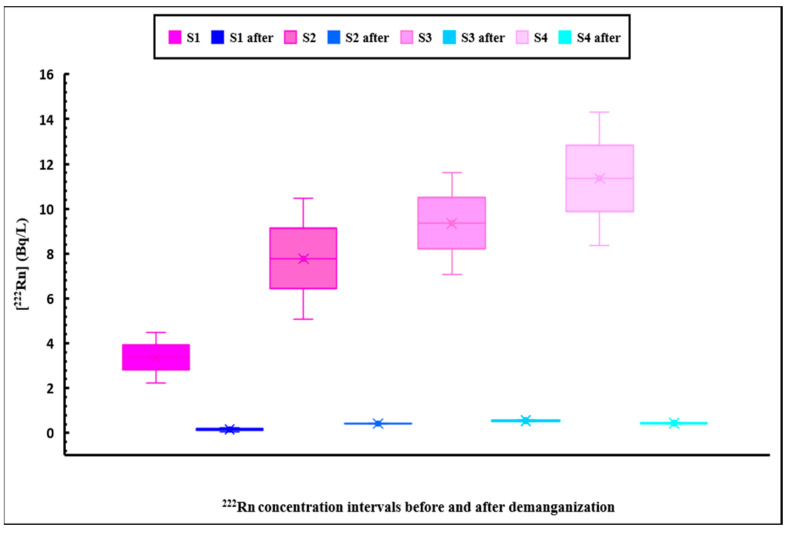
^222^Rn activity concentrations (Bq/L) before and after demanganization treatment.

Figure 6 shows the ^222^Rn concentrations before and after demanganization treatment for each of the four groundwater samples. The process under discussion reduces ^222^Rn concentrations for all the studied samples by about 20-fold on average.

#### 3.2.2. Fe^2+^ and Mn^2+^ Concentrations Before and After the Demanganization Treatment

The process of demanganization treatment is, in many cases, used if the water is intended to be used as a source of drinking water because the iron and manganese are regulated secondary drinking water species. The reduction in these contaminants is to improve the aesthetic quality of the final water product, as it is especially necessary for the improvement of the taste, smell, and color.

The raw water iron concentrations varied widely during the study, ranging from 3.95 ± 0.30 to 8.42 ± 0.40 mg/L, with a mean of 6.08 ± 0.35 mg/L (Table 3). Regarding the treated water, iron concentrations ranged from 0.35 ± 0.02 to 1.15 ± 0.04 mg/L, with a mean of 0.80 ± 0.03.

**Figure 7:**
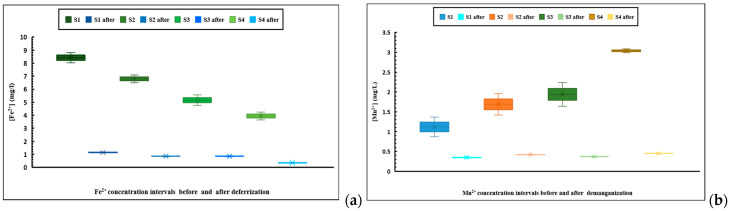
Fe^2+^ (**a**) Mn^2+^ (**b**) concentrations before and after treatment in the samples studied.

For the manganese levels in the raw water, these ranged from 1.12 ± 0.25 to 3.09 ± 0.05 mg/L, with a mean of 2.10 ± 0.22 mg/L, as for the treated water levels, they ranged from 0.35 ± 0.01 to 0.45 ± 0.01 mg/L, with a mean of 0.40 ± 0.01 mg/L (Table 3). As can be observed from Figure 7a,b and Table 4, the highest reduction for iron is for sample 1, since the highest reduction for manganese is for sample 4.

**Figure 8:**
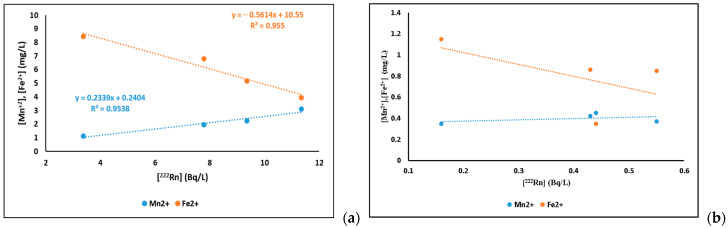
Correlation between ^222^Rn, Mn^2+^, and Fe^2+^ concentrations before (**a**) and after (**b**) treatment process.

As can be seen in Figure 8a, there is a strong correlation between the concentrations of ^222^Rn and Mn^2+^ before treatment, as both concentrations are increasing. For Fe^2+^ concentrations, these are decreasing as the ^222^Rn concentrations have higher values. The same tendency seems to occur after treatment, as shown in Figure 8b; however, the Mn^2+^ concentrations have relatively close values, which appear to remain approximately constant around a value of 0.4.

#### 3.2.3. Ammonium and Nitrate Concentrations Before and After the Demanganization Treatment

**Figure 9:**
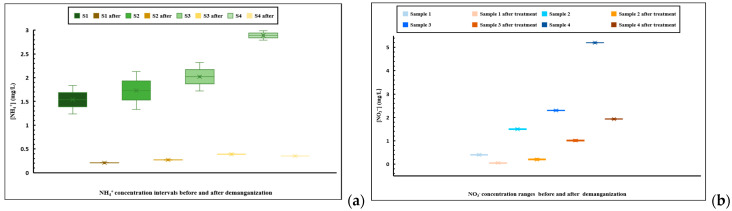
Ammonium (**a**) and NO_3_^−^ (**b**) concentrations before and after the treatment process.

Ammonium in the raw water ranged from 1.54 ± 0.30 to 2.89 ± 0.10 mg/L, with a mean of 2.04 ± 0.28 mg/L. Ammonium in the treated water ranged from 0.210 ± 0.003 to 0.390 ± 0.006 mg/L, with a mean of 0.305 ± 0.005 mg/L. The approximately 85% reduction performance level was achieved on average for all raw/treated sample pairs. All raw and treated water samples had nitrate concentrations ranging from 0.40 ± 0.04 to 5.20 ± 0.02 mg/L and from 0.05 ± 0.04 to 1.93 ± 0.03 mg/L after treatment, with means of 2.35 ± 0.03 mg/L and 0.80 ± 0.04 mg/L, respectively. The ammonium concentrations in all the groundwater samples studied could indicate an influence of fertilizers used in agricultural activities or the presence of domestic sewage in groundwater. The reduction in ammonium and nitrate concentrations is similar to that of manganese, with the highest reduction for sample 4, as can be observed in Figure 9a,b.

Following the treatment process, the removal percentage of the species studied in all the water studied was high, with an average of approximately 95% for ^222^Rn, 80% for Mn^2+^, 87% for Fe^2+^, 85% for NH_4_^+^, and 73% for NO_3_^−^ (Table 5). The removal percentage for ^222^Rn and NH_4_^+^ did not vary that much between samples, yet for the other species, significant differences were observed. For the first two samples, which had lower pH levels (6.2–6.3), Mn^2+^ and Fe^2+^ showed a lower removal percentage, while NO_3_^−^ had the highest removal percentage. Regarding the removal percentage of Fe^2+^ and NO_3_^−^ in sample 3, they had a lower value, and that might be because in this sample, the pH had an increased value (6.9), and the Fe^2+^ concentration had an average value in this sample. For the last sample, in which the pH had the highest value (7.8), the removal percentage for all the species was highest, except NO_3_^−^.

**Figure 10:**
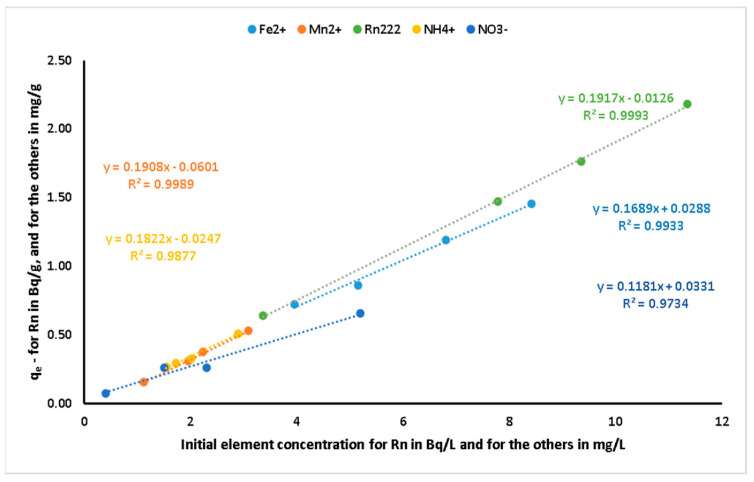
Removal of the Mn^2+^, Fe^2+^, nitrogen-containing species and ^222^Rn in the samples studied.

The variation functions for equilibrium sorption capacities are shown in Figure 10. The initial concentration of species affects the sorption capacity of the MnO_2_ sand. At the beginning of the process, the concentration of the species studied and the MnO_2_ sand binding sites have a linear relationship due to many vacancies on the surface of the sorbent. If the initial concentration increases, the mass transfer resistance between MnO_2_ sand and the species solution becomes smaller because of the increased driving force, resulting in an increased adsorption capacity. However, these vacant sites are gradually reduced and entirely occupied by various species, leading to a sharp downfall in adsorption. Therefore, optimization research for initial species concentration is indispensable when selecting a suitable sorbent material for environmental pollutants.

From Figure 10, the unit adsorption capacities, q_e_, values for ^222^Rn and the other species are increasing with increasing initial element concentration. The presence of coexisting ions produces competitive sorption processes with Mn^2+^. Fe^2+^ and NH_4_^+^ ions, present in the matrices, also influence the process due to the higher probability of coexisting with manganese in groundwater. When NH_4_^+^ ions and Mn^2+^ coexist, the removal effect of both is adversely affected [12]; however, this study did not show the same behavior, as Mn^2+^ initial concentration increases, its removal rate also increases, while for NH_4_^+^, as the initial concentration increases, the removal rate remains the same percentage. For Fe^2+^, as the initial concentration decreases, the removal rate increases. The removal of NO_3_^−^ seems to be affected especially by the pH of water samples.

## 4. Conclusions

In this study, the simultaneous removal of Mn^2+^, Fe^2+^, nitrogen-containing species, and ^222^Rn was investigated during water treatment using manganese oxide sand in four groundwater samples. After the treatment, it was observed that the concentration of all mentioned species, including ^222^Rn, was drastically reduced by about 95.00%, 79.06%, 87.08%, 84.83%, and 73.28% for ^222^Rn, Mn^2+^, Fe^2+^, NH_4_^+^, and NO_3_^−^, respectively. The removal rate remained stable for all four samples, between 94.12% and 96.12%, whereas Mn^2+^ and NO_3_^−^ showed high variability, between 68.75% and 85.44% and 56.09% and 87.50%, respectively.

Removal efficiency of ^222^Rn was not influenced by the presence of other ions such as iron and manganese, while the removal rate was higher than 94% on average, for all four samples. Understanding the adsorption mechanism using manganese oxide sand is an essential and complex process and can directly imply improvements in the feasibility of this process through different conditions and designs.

Sources of ^222^Rn activity in the groundwater are mainly related to the rock–water interactions in the aquifers, but anthropogenic influences might play a part too. This investigation will be continued in order to clarify the mechanism of ^222^Rn removal and its optimum experimental conditions.

## Figures and Tables

**Table 1:** Possible attributions of the spectral bands.

Possible Attribution	Wavenumber (cm^−1^)	
	Before Adsorption	After Adsorption
Si-O-Si	1164	-
Si-O-Si	1091	1086
O–Si–O	1035	1036
Si-O-Si	1009	1010
Si–O–M	936	-
Si–O–M	912	915
O–Si–O	797	796
O–Si–O	776	778
O–Si–O	719	724
Mn-O	-	694
Mn-O	668	669
Mn-O	-	649
Mn-O	591	586
Fe-O	540	-
Mn-O-Mn	-	531
Fe-O	470	463
Fe-O	444	-
Mn-O	-	421
Fe-O	417	-
Fe-O	397	397
Fe-O	383	-
Fe-O	371	372

**Table 2:** Chemical composition of the groundwater samples studied.

Sample	pH	Dry Residue, 180 °C (mg/L)	Electrical Conductivity (µS/cm)	Ca^2+^ (mg/L)	Mg^2+^, (mg/L)	Na^+^, (mg/L)	HCO_3_, (mg/L)	SO_4_^2−^, (mg/L)	Cl^−^ (mg/L)	Zn^2+^, (mg/L)
1	6.3	1080	1750	301	82	9.3	1319	29.0	16.6	1.2
2	6.2	745	990	144	25	3.2	517	8.0	5.4	1.7
3	6.9	634	1060	82	21	143.0	880	14.9	3.5	7.8
4	7.8	278	600	54	30	76.5	451	11.2	2.0	6.4

**Table 3:** Radon, anion, and cation concentrations before and after removal by MnO_2_ sand.

Before Deferrization/Demanganization								After Deferrization/Demanganization						
S	pH	Dry Residue 180° mg/L	^222^Rn, (Bq/L)	Mn^2+^, (mg/L)	Fe^2+^, (mg/L)	NH_4_^+^, (mg/L)	NO_3_^−^, (mg/L)	pH	Dry Residue 180° mg/L	^222^Rn, (Bq/L)	Mn^2+^, (mg/L)	Fe^2+^, (mg/L)	NH_4_^+^, (mg/L)	NO_3_^−^, (mg/L)
1	6.3	1080	3.37 ± 1.12	1.12 ± 0.25	8.42 ± 0.40	1.54 ± 0.30	0.40 ± 0.04	7.3	338	0.16 ± 0.09	0.35 ± 0.01	1.15 ± 0.04	0.210 ± 0.003	0.05 ± 0.04
2	6.2	745	7.78 ± 1.59	1.96 ± 0.27	6.80 ± 0.30	1.73 ± 0.40	1.50 ± 0.03	7.2	304	0.43 ± 0.01	0.42 ± 0.01	0.86 ± 0.03	0.270 ± 0.006	0.20 ± 0.05
3	6.9	634	9.35 ± 2.28	2.24 ± 0.30	5.15 ± 0.40	2.02 ± 0.30	2.30 ± 0.02	8.1	181	0.55 ± 0.03	0.37 ± 0.01	0.85 ± 0.03	0.390 ± 0.006	1.01 ± 0.05
4	7.8	278	11.35 ± 2.97	3.09 ± 0.05	3.95 ± 0.30	2.89 ± 0.10	5.20 ± 0.02	8.0	168	0.44 ± 0.02	0.45 ± 0.01	0.35 ± 0.02	0.350 ± 0.003	1.93 ± 0.03

**Table 4:** Calculated equilibrium adsorption capacity of MnO_2_ sand of the studied species.

	q_e_ Rn^222^	q_e_ Mn^2+^	q_e_ Fe^2+^	q_e_ NH_4_^+^	q_e_ NO_3_^−^
Sample	Bq/g	mg/g	mg/g	mg/g	mg/g
1	0.64	0.15	1.45	0.27	0.07
2	1.47	0.31	1.19	0.29	0.26
3	1.76	0.37	0.86	0.33	0.26
4	2.18	0.53	0.72	0.51	0.65

**Table 5:** Removal percentage of the studied species.

Sample	pH	^222^Rn Removal Rate (%)	Mn^2+^ Removal Rate (%)	Fe^2+^ Removal Rate (%)	NH_4_^+^ Removal Rate (%)	NO_3_^−^ Removal Rate (%)
1	6.3	95.25	68.75	86.34	86.36	87.50
2	6.2	94.47	78.57	87.35	84.39	86.67
3	6.9	94.12	83.48	83.50	80.69	56.09
4	7.8	96.12	85.44	91.14	87.89	62.88
Mean	6.8	95.00	79.06	87.08	84.83	73.28

## Data Availability

The original contributions presented in this study are included in the article. Further inquiries can be directed to the corresponding author.
